# Targeted Therapy in the Palliative Setting of Colorectal Cancer—Survival and Medical Costs

**DOI:** 10.3390/cancers15113022

**Published:** 2023-06-01

**Authors:** Kamuran Inci, Bengt Nilsson, Lars Ny, Ulf Strömberg, Nils Wilking, Stefan Lindskog, Daniel Giglio

**Affiliations:** 1 Department of Surgery, Institute of Clinical Sciences, Sahlgrenska Academy, University of Gothenburg, 40530 Gothenburg, Sweden; kamuran.inci@regionhalland.se (K.I.);; 2 Department of Surgery, Halland Hospital Varberg, Region Halland, 43237 Varberg, Sweden; 3 Department of Oncology, Institute of Clinical Sciences, Sahlgrenska Academy, University of Gothenburg, 40530 Gothenburg, Sweden; lars.ny@oncology.gu.se; 4 Department of Oncology, Sahlgrenska University Hospital, 41345 Gothenburg, Sweden; 5 Department of Research and Development, University of Gothenburg, Region Halland, 40530 Gothenburg, Sweden; 6 Department of Oncology-Pathology, Karolinska Institute, 17176 Stockholm, Sweden; nils.wilking@ki.se

**Keywords:** colorectal cancer, palliative, bevacizumab, EGFr, survival

## Abstract

**Simple Summary:**

Targeted therapy targeting the EGFr and vascular endothelial growth factor (VEGF) is often combined with chemotherapy in palliative treatment of metastatic colorectal cancer. The aim of this population-based cohort study was to examine factors influencing treatment decisions for targeted therapy and their effect on overall survival in palliative metastatic colorectal cancer. The medical costs of targeted therapy if introduced early or late in the palliative setting were also assessed. The results showed no effect of high medical costs on overall survival for patients with metastatic colorectal cancer. The medical costs are high and especially so if introduced early in the palliative setting. We suggest that targeted therapy be saved for later lines of therapy in patients with palliative colorectal cancer.

**Abstract:**

(1) Background: Targeted therapy is used alone or together with chemotherapy in metastatic colorectal cancer. The aim of this study was to assess overall survival and medical costs in a cohort of patients with metastatic colorectal cancer. (2) Methods: Demographic and clinical characteristics of 337 patients and pathological data of colorectal tumors were retrospectively collected in this population-based study. The overall survival and medical costs for patients receiving chemotherapy plus targeted therapy were compared with those for patients receiving chemotherapy only. (3) Results: Patients administered chemotherapy plus targeted therapy were less frail and had more often RAS wild-type tumors but had higher CEA levels than patients receiving chemotherapy only. No prolonged overall survival could be observed in patients receiving palliative targeted therapy. The medical costs for patients undergoing treatment with targeted therapy were significantly higher than for patients treated only with chemotherapy; they were especially higher in the group receiving targeted therapy early than late in the palliative setting. (4) Conclusions: The use of targeted therapy in metastatic colorectal cancer leads to significantly higher medical costs when used early in the palliative setting. No positive effects of the use of targeted therapy could be observed in this study; therefore, we suggest that targeted therapy be used in later lines of palliative therapy in metastatic colorectal cancer.

## 1. Introduction

After prostate and breast cancer, colorectal cancer is the most common form of cancer in Western countries, with about 500,000 people diagnosed in Europe and about 148,000 people diagnosed in the United States annually. Tumors located in the left colon are different from tumors located in the right colon with respect to tumor phenotypes and clinical treatment outcomes [1]. Tumors derived from the right colon are more often mutated in Kirsten rat sarcoma viral oncogene homolog (KRAS) and V-raf murine sarcoma viral oncogene homolog B1 (BRAF), are more often deficient mismatch repair (dMMR)/microsatellite instability-high (MSI-H) tumors, and generally have a worse prognosis than tumors situated in the left colon, where activation of the epidermal growth factor receptor (EGFr) is more common instead [2,3,4].

Combinations of chemotherapy, including fluoropyrimidines, oxaliplatin, and irinotecan, are used in neoadjuvant and adjuvant settings for patients with locally advanced colorectal cancer with lymph node metastases or when metastasectomy is performed [5,6]. The same set of chemotherapy treatments is also used in palliative treatment. The use of targeted therapy in metastatic colorectal cancer is increasing; hence, more knowledge of survival and costs is of potential interest. Targeted therapy including drugs targeting the EGFr and vascular endothelial growth factor (VEGF) is often combined with chemotherapy in the palliative setting. The EGFr-targeted drug cetuximab demonstrates clinical efficacy in first- [7,8], second- [9], and third-line [10] treatment alone or in combination with chemotherapy in patients with RAS wild-type metastatic colorectal cancer. Adding the EGFr-targeted drug panitumumab to chemotherapy augments progression-free survival (PFS) in first- [11] and second-line [12] settings and is superior alone over the best supportive care for patients with metastatic colorectal cancer who have progressed on oxaliplatin- and irinotecan-containing regimens [13]. Bevacizumab, targeting the VEGF, may be introduced as first-, second-, or third-line therapy combined with chemotherapy in metastatic colorectal cancer [8,14]. In many countries, EGFr- and VEGF-targeted therapies are introduced in the first-line setting of metastatic colorectal cancer [15]. However, few randomized trials have assessed whether targeted therapy affects survival differently if it is introduced in the first line or in later lines of therapy. Cost-effectiveness is suggested to be low when bevacizumab together with chemotherapy is used in the first-line treatment of metastatic colorectal cancer [16,17].

The aim of this study was to examine factors influencing treatment decisions for targeted therapy and their effect on overall survival in palliative metastatic colorectal cancer. The medical costs of targeted therapy if introduced early or late in the palliative setting were also assessed.

## 2. Materials and Methods

**Figure 1:**
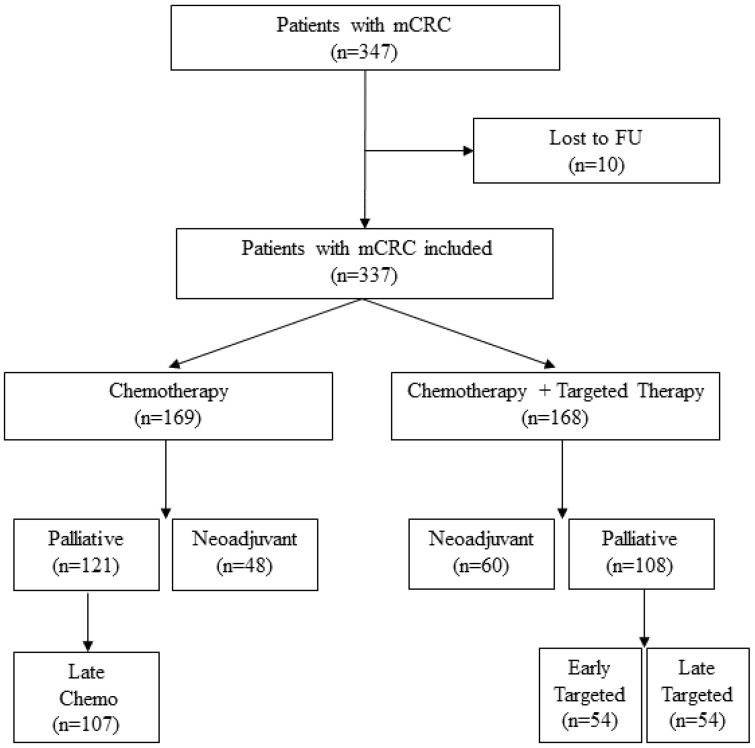
Flowchart of patients, with an illustration of the subdivisions in the palliative cohort.

This population-based cohort study included all 347 patients diagnosed with metastatic colorectal cancer at any time from 2008 to 2018 in Region Halland, Sweden, which has a population of 340,000. The patients were identified through their electronic medical records. It is easy to follow-up patients in this region due to low migration, the regional common medical record system, and the Swedish national personal code number being available. Patients who agreed to be treated with chemotherapy and/or targeted therapy were included in the study. All patients were discussed at a multidisciplinary tumor board, in which oncologists, surgeons, pathologists, and radiologists participated, where either the palliative or the neoadjuvant therapeutic approach was decided for each patient. Neoadjuvant patients were administered chemotherapy and/or targeted therapy with the intent to perform surgery of liver metastases (83%), the primary tumor (11%), or lung metastases (6%). Clinical and demographic data were recorded, as well as data from pathology reports. In total, 10 patients were excluded due to incomplete information (Figure 1).

The demographic and clinical characteristics of the 337 patients and pathological data of the tumors were registered in a database (Table 1). The date of metastatic colorectal cancer diagnosis was considered the point in time for the biopsy of the primary tumor when metastases were detected on CT scans. When biopsies were not taken, the date of metastatic colorectal cancer was considered the date when the CT scans detected metastases. The patients were followed from the detection of metastatic disease until death or the cut-off date of 31 December 2019.

Patients diagnosed with ICD C18.0–C18.4 were categorized in the right-sided colorectal tumor group, patients diagnosed with ICD C18.5–C18.7 and C19.9 were categorized in the left-sided colorectal tumor group, and patients diagnosed with ICD C20.9 were categorized in the rectal tumor group. The patients were grouped according to whether they had been exposed to chemotherapy plus targeted therapy or to chemotherapy only. Metastases to the liver, lungs, peritoneum, lymph nodes, and other organs in the abdominal cavity, skeleton, and brain were registered in the database. Whether a patient had diabetes mellitus, hypertonia, or cardiovascular disease (stroke, myocardial infarction, and heart fibrillation disease) was also recorded in the database. Each patient’s performance status according to the Eastern Cooperative Oncology Group (ECOG) at the time of diagnosis was also registered. Since not all patients were evaluated at this point, patients were given an ECOG PS score of 0 if their medical records did not indicate any symptoms associated with metastatic colorectal cancer and an ECOG PS score of 1 if their medical records indicated symptoms associated with metastatic colorectal cancer.

### 2.1. Chemotherapy and Targeted Therapy

Targeted therapy was defined as therapies targeting the EGFr (cetuximab and panitumumab) or VEGF (bevacizumab). The total number of treatment cycles for each chemotherapy combination and targeted therapy is shown in Supplementary Table S1. Each treatment cycle was defined as the standard treatment for each type of chemotherapy or combination therapy. Individual adjustments with reduced doses of standard therapy were not taken into consideration.

### 2.2. Classification of Patients

The cohort was subdivided into patients treated with either chemotherapy plus targeted therapy or chemotherapy only and grouped as neoadjuvant or palliative at the date of diagnosis of metastatic colorectal cancer (baseline). The median day for starting targeted therapy (i.e., EGFr-targeted therapy and bevacizumab) from baseline was then calculated. To assess the effect of targeted therapy on overall survival and the medical costs if introduced early or late in the palliative setting, the palliative group was divided into the early targeted therapy group (early targeted) if targeted therapy had started by the median day and the late targeted therapy group (late targeted) if targeted therapy had started after the median day (index day). Similar segmentation was performed for the palliative chemotherapy group for comparison with patients in the late chemotherapy group (late chemo) who were alive from the index day. The classification of the groups is demonstrated in Figure 1.

### 2.3. Costs of Chemotherapy and Targeted Therapy

The average medication costs of chemotherapy and targeted therapy are shown in Table 2. The costs for a body surface area (BSA) of 1.8 m^2^ (the representative BSA for patients with cancer [18]) were calculated for capecitabine (4600 mg daily; daily dose of 2500 mg/m^2^ for 14 days per treatment cycle of 21 days), trifluridine/tipiracil (120 mg daily; daily dose of 70 mg/m^2^ for 10 days per treatment cycle of 28 days), and tegafur/gimeracil/oteracil (110 mg daily; daily dose of 60 mg/m^2^ for 14 days per treatment cycle of 21 days; Table 2). For regorafenib, the costs of a dose of 160 mg daily for 21 days per treatment cycle of 28 days were calculated. The fixed costs were then multiplied by the number of treatments of each chemotherapy and targeted therapy administered to each patient. Individual adjustments with reduced doses of standard therapy were not taken into consideration. In addition, additional treatment costs except for medical costs were not taken into consideration in this analysis. All costs are expressed in euros.

### 2.4. Statistical Analysis

The chi-square test was performed to compare categorical variables, and the Mann–Whitney U test was performed to compare continuous variables between the groups. Cox regression univariate and multivariate analyses were performed to identify factors predicting overall survival, and crude hazard ratios (CHRs) and adjusted hazard ratios (AHRs) with 95% confidence intervals (CIs) were estimated. Targeted therapy constituted a time-dependent variable in the Cox regression analyses. *p*-Values less than 0.05 were considered statistically significant. The mean value ± standard deviation (SD) is given in the text. Our statistical consultant, author U.S., controlled the statistics in the study. IBM SPSS Statistics version 26 (IBM Corp., Armonk, NY, USA) and GraphPad Prism program 9.1.0 (GraphPad Software, Inc., San Diego, CA, USA) were used to analyze the data.

## 3. Results

### 3.1. Characteristics of Patients Treated with and without Targeted Therapy

Of a total of 337 patients with metastatic colorectal cancer, 108 patients (32%) were considered curable and were administered neoadjuvant treatment, while 229 patients (68%) were considered palliative. No differences in overall survival were observed between recurrent and de novo metastatic colorectal cancer. The patients administered targeted therapy were younger than the patients receiving chemotherapy only, i.e., 61.3 ± 9.6 years vs. 67.1 ± 10.7 years, respectively (*n* = 168 and *n* = 169, respectively; *p* < 0.05), and had fewer comorbidities. The RAS was more often wild type in the targeted therapy group than in the chemotherapy group (Table 1). Moreover, carcinoembryonic antigen (CEA) levels at baseline were higher in patients receiving targeted therapy compared to patients receiving chemotherapy only (Table 1). No differences between the groups were observed regarding gender proportions, performance status, the location of the primary tumor, the number of metastases, surgery of the primary tumor or liver, and the proportion of patients considered neoadjuvant at the point in time for metastatic colorectal cancer. In patients with rectal cancer considered neoadjuvant and palliative, 83% and 67% underwent radiotherapy against the rectal tumor, respectively.

### 3.2. Characteristics of the Palliative Subgroups

The early targeted group consisted of younger patients with fewer comorbidities compared to patients receiving chemotherapy only (Table 3). Only 12% of patients in the palliative chemotherapy group died during the first 131 days; therefore, the late chemo group constituted 88% of the whole palliative chemotherapy group. Like the early targeted and palliative groups, patients in the late targeted group were younger and had a better performance status than patients receiving chemotherapy only (Table 3). No differences were observed between the early targeted and palliative groups and between the late targeted and late chemo groups regarding gender proportions, CEA levels, the location of the primary tumor, the number of metastatic sites, or the percentage undergoing surgery of the primary tumor or liver or RAS mutations.

### 3.3. Chemotherapy and Targeted Therapy

**Figure 2:**
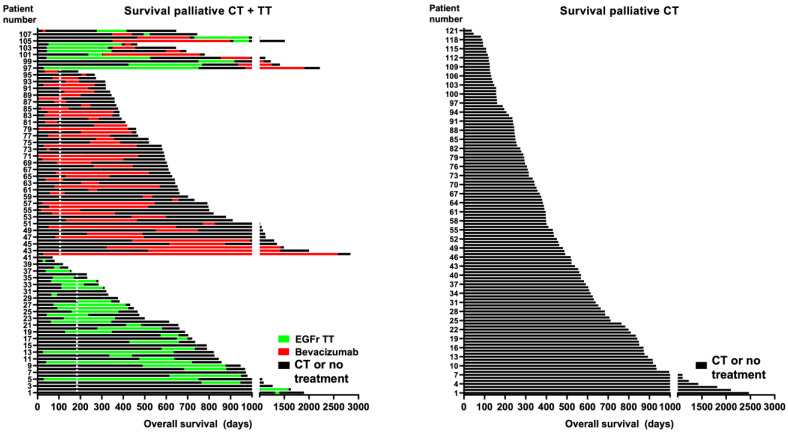
Overall survival in days of patients in the palliative targeted therapy group, where green lines indicate the time window for exposure to EGFr-targeted therapy, red lines indicate the time window for exposure to bevacizumab, and black lines indicate the time window for exposure to either chemotherapy or no treatment. The dotted vertical line indicates the median days before the introduction of bevacizumab or EGFr-targeted therapy.

The total number of chemotherapy and targeted therapy cycles is visualized in Supplementary Table S1. The chemotherapy plus targeted therapy group received a significantly higher number of chemotherapy cycles including fluoropyrimidines, irinotecan, and oxaliplatin combinations compared to patients receiving chemotherapy only (see Supplementary Table S1). In the targeted therapy group, 51% of patients received bevacizumab, 35% received EGFr-targeted therapy, and 14% were treated with both bevacizumab and EGFr-targeted therapies during their lifetime. Patients receiving EGFr-targeted therapy were treated with panitumumab (Vectibix^®^) and cetuximab (Erbitux^®^) in 80% and 20% of cases, respectively. Patients treated with bevacizumab (Avastin^®^) received on average 13.0 ± 12.5 cycles (*n* = 83), and patients treated with EGFr-targeted therapy received on average 9.1 ± 9.0 cycles (*n* = 62). In addition, 23 patients received both bevacizumab (Avastin^®^) and EGFr-targeted therapy during their lifetime. Bevacizumab was given alone in 2% of cases or combined with a single fluoropyrimidine agent (20%), an irinotecan-based doublet regimen (61%), an oxaliplatin-based doublet regimen (16%), or triplet chemotherapy (FOLFOXIRI; 2%). EGFr-targeted therapies were given alone in 21% of cases or combined with a single fluoropyrimidine agent (11%) or irinotecan alone (10%), an irinotecan-based doublet regimen (46%), an oxaliplatin-based doublet regimen (12%), or triplet chemotherapy (FOLFOXIRI; 1%). In palliative patients exposed to bevacizumab, the median day until the introduction of bevacizumab was 102 days, while the corresponding figure for patients exposed to EGFr-targeted therapy was 182 days (Figure 2).

### 3.4. Administration Patterns of Targeted Therapy

The median number of days to initiate targeted therapy in the palliative group (*n* = 229) was 131.5 days from the day of diagnosis of metastatic colorectal cancer. Patients in the early targeted and late targeted groups were treated on average with 10.6 ± 9.6 and 8.2 ± 7.2 bevacizumab treatments, respectively, and on average with 15.6 ± 11.6 and 11.1 ± 14.1 EGFr treatments, respectively. In the early targeted group, 62.5% of patients were introduced to targeted therapy in the first line, 20.0% in the second line, 7.5% in the third line, 5.0% in the fourth line, and 5.0% in later lines of therapy. In the late targeted group, 11.6% of patients were introduced to targeted therapy in the first line, 16.3% in the second line, 37.2% in the third line, 23.3% in the fourth line, and 11.6% in later lines of therapy.

### 3.5. Overall Survival and Characteristics of Targeted Therapies

Time-dependent covariate Cox regression analyses compensating for immortal bias showed no positive effects of bevacizumab on overall survival in the palliative setting of metastatic colorectal cancer. Patients treated with EGFr-targeted therapy had worse overall survival from the time that it was introduced (Table 4).

Among the patients considered palliative, an impaired performance status at baseline, the presence of liver metastases, and an unknown RAS mutation status compared to RAS wild-type tumors were factors associated with worse overall survival (Table 4). Left-sided colorectal and rectal cancers were associated with better overall survival compared to right-sided colorectal cancers. High CEA levels were associated with worse overall survival in the univariate analysis and tended to do so in the multivariate analysis (Table 4).

### 3.6. Medical Costs of Targeted Therapy

Patients treated with targeted therapy in the neoadjuvant setting had 18.7 times higher total costs and 9.8 times higher annual costs than patients treated with chemotherapy only (*p* < 0.001; Table 5). For palliative metastatic colorectal cancer, the corresponding increases in costs were 13.8 and 8.7 times, respectively (*p* < 0.001; Table 5). The annual costs were significantly higher in the early targeted group than in the late targeted group.

## 4. Discussion

Targeted therapy was offered to healthier patients in this study without survival benefits compared to only chemotherapy in palliative colorectal cancer. The medical costs of targeted therapy were high, especially when introduced early in the palliative setting. This study provides no support for using targeted therapy early in palliative metastatic colorectal cancer. While bevacizumab had no effects on overall survival, patients treated with even EGFr-targeted therapy had worse overall survival from the time it was introduced. This finding, however, does not likely mean that EGFr treatment affects overall survival negatively; instead, it is more plausible that this was due to the effect of when EGFr-targeted therapy was introduced, i.e., EGFr-targeted therapy was often introduced in later lines of therapy compared to bevacizumab and in late stages. Studies show that therapies targeting the VEGF and EGFr may be effective if introduced in later lines of therapy in the palliative setting of metastatic colorectal cancer [10,13,19,20,21,22]. A meta-analysis of the use of EGFr-targeted therapy also suggested that saving EGFr-targeted monotherapy until the third line is an effective option in wild-type metastatic colorectal cancer [21].

In line with previous studies, a poor performance status [23,24], high CEA levels at baseline [25,26], right-sided colorectal cancer [23,27], and the presence of liver metastases [24] were correlated with shorter overall survival. Of note, we acknowledge that the effects of performance status may be under-estimated in our study, since when information about ECOG PS was lacking and symptoms were present, the ECOG PS score was set to no more than 1. We found that an unknown RAS mutation status negatively affects overall survival in patients with palliative colorectal cancer. The prognostic significance of RAS mutation in colorectal cancer varies between studies; however, a meta-analysis showed that overall survival in patients with RAS codon 13 mutations is worse compared to RAS wild-type tumors [28]. When survival was compared between patients with RAS codon 13 mutations and patients with RAS wild-type tumors treated with EGFr-targeted therapy, survival tended to be better in the latter group; however, statistical significance was not attained [28]. In our group of patients with palliative colorectal cancer with an unknown RAS mutation status (with the majority diagnosed at the end of the 2000s), the patients were offered less intensive chemotherapy, were seldom offered targeted therapy, and were older compared to patients being tested for RAS, which could have contributed negatively to overall survival.

Age was not a predictor of overall survival in this study. Conflicting results on whether age is a predictor of overall survival in metastatic colorectal cancer have been reported in previous studies and are most likely dependent on how the age groups are defined, whether age is assessed as a categorical or a continuous variable, and the number of patients in the respective age groups [26,29,30]. Large-scale studies show that the risk of death seems to be highest among the youngest and oldest patients compared to middle-aged patients with metastatic colorectal cancer [30]. Our study may have been underpowered in the youngest and oldest age groups.

Palliative and neoadjuvant patients treated with only chemotherapy were older, had more comorbidities, and consisted of a higher proportion with an unknown RAS mutation status, but they had lower CEA levels than patients treated with targeted therapy. The performance status was worse at baseline in patients not being administered targeted therapy in later lines of therapy.

The chemotherapy plus targeted therapy group was subdivided into two subgroups (early targeted and late targeted) in relation to the median day (index day) of the introduction of targeted therapy. The control groups, treated with chemotherapy only, consisted of the whole palliative group, and the late chemo group comprised all patients alive after the index day. We know the performance status when targeted therapy was introduced but not the health condition of the control group alive from the index day. However, the index day was only 131.5 days from baseline, and only 12% of patients in the control group succumbed by the index day, showing that the early and late targeted groups consisted of similar cohorts of patients. Patients treated with targeted therapy also underwent longer treatment periods of chemotherapy compared to patients treated with chemotherapy only. The effect of targeted therapy and chemotherapy on overall survival was therefore difficult to fully distinguish. Younger patients were more often treated with targeted therapy. Franchi et al. [31] showed that chemotherapy combined with targeted therapy in the first-line treatment of metastatic colorectal cancer significantly increases costs without affecting overall survival. These findings are in line with our study; the costs of oncological treatments were higher in the early targeted group compared to the late targeted group. Medical costs are a major factor that significantly affect overall costs; therefore, we did not assess other health care costs besides medical costs. The costs of targeted therapy and chemotherapy are equivalent in Western countries with roughly equivalent populations. This can differ in low-income countries due to discounts, but this study compared the differences between the costs of targeted therapy and chemotherapy, which makes the results interesting even for stakeholders in low-income countries. Our results indicate that targeted therapy may be more cost-efficient if introduced in later lines of therapy. However, the cost of targeted therapy may drop in the future for Erbitux^®^, Vectibix^®^, and Avastin^®^ since the patents have expired [32,33].

## 5. Conclusions

In conclusion, targeted therapy has no positive effects on overall survival in the palliative setting in patients with metastatic colorectal cancer. The medical costs are high and especially so if introduced early in the palliative setting. We suggest that targeted therapy be saved for later lines of therapy in patients with palliative colorectal cancer.

## Figures and Tables

**Table 1:** Baseline demographics and clinical data of patients treated with chemotherapy only and patients treated with chemotherapy and targeted therapy.

	Chemotherapy (*n* = 169) *n* (%)	Targeted Therapy (*n* = 168) *n* (%)
Gender		
Male	99 (59)	99 (59)
Female	70 (41)	69 (41)
Age (mean ± SD) *	67.1 ± 10.7	61.3 ± 9.6
ECOG PS		
0	68 (40)	81 (48)
1	89 (53)	83 (50)
2, 3	12 (7)	4 (2)
Comorbidity		
Cardiovascular disease *	42 (25)	19 (11)
Hypertonia *	64 (38)	46 (27)
Diabetes mellitus	21 (12)	16 (10)
Location of the primary tumor		
Right colon	46 (27)	45 (27)
Left colon	64 (38)	61 (36)
Rectum	57 (34)	57 (34)
Multiple tumors	2 (1)	5 (3)
Metastatic sites		
1	90 (53)	89 (53)
2	58 (34)	52 (31)
≥3	21 (12)	27 (16)
Liver metastases	123 (73)	129 (77)
Lung metastases	60 (36)	50 (30)
CEA at baseline (mean ± SD) *	106 ± 289 ng/mL (*n* = 132)	171 ± 363 ng/mL (*n* = 133)
Liver surgery	31 (18)	29 (17)
RAS *		
Wild type	64 (38)	90 (54)
Mutated	65 (38)	61 (36)
Unknown	40 (24)	17 (10)
Surgery of the primary tumor	99 (59)	101 (60)
Treatment intention		
Palliative	121 (72)	108 (64)
Neoadjuvant	48 (28)	60 (36)

* Indicates *p* < 0.05. SD = standard deviation.

**Table 2:** Costs of chemotherapy and targeted therapy in euros.

Group	Average Costs per Treatment Cycle (EUR)
Bevacizumab	1351
Cetuximab	1353
Panitumumab	1619
5-Fluorouracil	83
Irinotecan	77
Oxaliplatin	68
Capecitabine	105
Trifluridine/tipiracil	2200
Tegafur/gimeracil/oteracil	306
Regorafenib	2174

**Table 3:** Clinical and tumor characteristics of the palliative cohort with the whole chemotherapy group and the late chemo (late palliative chemotherapy) group, and the chemotherapy plus targeted therapy group subdivided into early targeted and late targeted groups. The early targeted group was compared with the chemotherapy group, the late targeted group, and the late chemo group.

	Chemotherapy (*n* = 121) *n* (%)	Early Targeted (*n* = 54) *n* (%)	Late Chemo (*n* = 107) *n* (%)	Late Targeted (*n* = 54) *n* (%)
Gender				
Male	71 (59)	31 (57)	64 (60)	33 (61)
Female	50 (41)	23 (43)	43 (40)	21 (39)
Age (mean ± SD)	68.5 ± 10.7	59.8 ± 9.8 ***	68.3 ± 10.7	64.7 ± 7.8 *
ECOG PS				
0	43 (36)	20 (37)	41 (38)	33 (61)
1	68 (56)	32 (59)	58 (54)	20 (37)
2, 3	10 (8)	2 (4)	8 (7)	1 (2)
Comorbidity				
Cardiovascular disease	29 (24)	5 (9)	28 (26)	8 (15)
Hypertonia	47 (38)	11 (20) *	44 (41)	18 (33)
Diabetes mellitus	15 (12)	5 (9) *	14 (13)	6 (11)
Location of the primary tumor				
Right colon	37 (31)	16 (30)	29 (27)	17 (31)
Left colon	42 (35)	17 (32)	40 (37)	21 (39)
Rectum	40 (33)	18 (33)	37 (35)	16 (30)
Multiple tumors	2 (2)	3 (6)	1 (1)	0 (0)
Metastatic sites				
1	52 (53)	26 (48)	48 (45)	26 (48)
2	50 (34)	21 (39)	44 (41)	21 (39)
3	17 (11)	5 (9)	13 (12)	5 (9)
4	2 (1)	2 (4)	2 (2)	2 (4)
5	0 (0)	0 (0)	0 (0)	3 (6)
Metastatic sites				
1	52 (53)	26 (48)	48 (45)	26 (48)
2	50 (34)	21 (39)	44 (41)	21 (39)
≥3	19 (16)	7 (13)	15 (14)	10 (19)
Liver metastases	84 (69)	40 (74)	73 (68)	41 (76)
Lung metastases	51 (42)	12 (22)	44 (41)	24 (44)
CEA at baseline (mean ± SD)	137 ± 334 ng/mL (n = 95)	214 ± 431 ng/mL (n = 46)	128 ± 331 ng/mL (n = 85)	140 ± 272 ng/mL (n = 46)
Liver surgery	31 (18)	29 (17)	5 (5)	3 (6)
RAS				
Wild type	51 (42)	30 (56)	41 (38)	28 (52)
Mutated	45 (37)	18 (33)	44 (41)	20 (37)
Unknown	25 (21)	6 (11)	22 (21)	6 (11)
Surgery of the primary tumor	55 (46)	24 (44)	52 (49)	31 (57)

* Indicates *p* < 0.05. *** Indicates *p* < 0.001. SD = standard deviation.

**Table 4:** Factors associated with overall survival in the palliative cohort. Targeted therapy constituted a time-dependent variable in the Cox regression analyses.

Palliative (*n* = 229)	Univariate Analysis		Multivariate Analysis	
	HR (95% CI)	*p*-Value	HR (95% CI)	*p*-Value
Age	1.00 (0.99–1.02)	0.58	1.70 (0.87–3.32)	0.12
Gender				
Female	Reference		Reference	
Male	1.04 (0.78–1.38)	0.79	0.94 (0.69–1.28)	0.68
ECOG PS				
0	Reference	<0.001	Reference	<0.001
1	1.66 (1.23–2.24)	<0.001	1.79 (1.27–2.52)	<0.001
2	1.62 (0.75–3.53)	0.22	2.94 (1.24–6.99	<0.05
3	7.31 (2.91–18.37)	<0.001	10.72 (3.79–30.3)	<0.001
Hypertonia				
No	Reference		Reference	
Yes	1.15 (0.85–1.55)	0.36	1.11 (0.78–1.58)	0.57
Cardiovascular disease				
No	Reference		Reference	
Yes	1.08 (0.76–1.55)	0.67	0.93 (0.61–1.41)	0.73
Diabetes mellitus				
No	Reference		Reference	
Yes	1.24 (0.81–1.91)	0.32	0.81 (0.51–1.30)	0.39
Tumor location		0.06		<0.001
Right colon	Reference		Reference	
Left colon	0.74 (0.53–1.05)	0.09	0.55 (0.38–0.79)	<0.01
Rectum	0.62 (0.44–0.88)	<0.01	0.45 (0.30–0.67)	<0.001
Multiple tumors	0.75 (0.30–1.88)	0.55	0.72 (0.28–1.89)	0.51
Liver metastases				
No	Reference		Reference	
Yes	1.65 (1.18–2.31)	<0.01	1.53 (1.06–2.19)	<0.05
Lung metastases				
No	Reference		Reference	
Yes	0.71 (0.53–0.94)	<0.05	0.80 (0.55–1.17)	0.25
No. of metastatic sites	1.04 (0.89–1.21)	0.64	1.11 (0.92–1.35)	0.27
RAS		0.19		<0.01
Wild type	Reference		Reference	
Mutated	1.14 (0.83–1.56)	0.43	1.27 (0.88–1.81)	0.20
Unknown	1.44 (0.97–2.12)	0.07	1.62 (1.30–3.09)	<0.01
Targeted therapy				
Chemotherapy	Reference	0.05	Reference	<0.05
Bevacizumab	0.89 (0.62–1.27)	0.52	0.81 (0.54–1.20)	0.30
EGFr	1.55 (1.05–2.30)	<0.05	1.74 (1.11–2.72)	0.05
Both therapies	1.49 (0.78–2.83)	0.23	1.70 (0.87–3.32)	0.12
CEA * n = 183	1.0009 (1.0004–1.0013)	<0.001	1.0006 (0.9999–1.0012)	0.06

* Due to missing values, univariate and multivariate analyses for the carcinoembryonic antigen (CEA) were calculated separately based on *n* = 183. HR = hazard ratio; CI = confidence interval.

**Table 5:** Costs of chemotherapy plus targeted therapy vs. chemotherapy alone for the neoadjuvant and palliative cohorts, respectively, and costs when targeted therapy was introduced early and late in palliative therapy (early and late targeted). The late chemotherapy subgroup is presented as a reference to the medical costs for the late targeted group.

Group	No. of Patients	Average Total Costs ± SD (EUR)	Average Annual Costs ± SD (EUR)
Neoadjuvant			
Chemotherapy + targeted therapy	60	22,150 ± 24,079 ***	7249 ± 4801 ***
Chemotherapy	48	1184 ± 712	737 ± 1188
Palliative			
Chemotherapy + targeted therapy	108	20,925 ± 17,480 ***	11,437 ± 6868 ***
Chemotherapy ^#^	120	1514 ± 1962	1322 ± 1351
Early targeted	54	23,982 ± 18,129 ^†^	15,190 ± 6569 ***
Late targeted	54	17,868 ± 16,407	7685 ± 4836
Late chemo	106	1668 ± 2037	1270 ± 1227

*** Indicates *p* < 0.001 between early targeted and late targeted groups. ^†^ Indicates *p* = 0.07 between early targeted and late targeted groups. ^#^ One patient was excluded due to missing data.

## Data Availability

Additional data are available upon request to the Institute of Clinical Sciences, Sahlgrenska Academy at the University of Gothenburg.

## Supplementary Materials

The following supporting information can be downloaded at https://www.mdpi.com/article/10.3390/cancers15113022/s1: Table S1: Chemotherapy and targeted therapy administered to the palliative cohort, with each cycle defined as the standard treatment for each type of chemotherapy.
