# Supplementary material for: Targeted Therapy in the Palliative Setting of Colorectal Cancer—Survival and Medical Costs
Source: Cancers (Basel). 2023 Jun 1;15(11):3022. doi: 10.3390/cancers15113022 (PMC10252087; doi:10.3390/cancers15113022)
Supplement: Supplementary file 1 [file cancers-15-03022-s001.zip › cancers-2359623-supplementary.pdf]

**Supplemental Table S1**. Chemotherapy and targeted therapy given in the palliative cohort (N=229) with each cycle was defined as standard treatment for each type of chemotherapy * indicates p<0.05.

| | **Chemotherapy** (n=169) | **Chemotherapy + Targeted Therapy** (N=168) |
|---|---|---|
| Nordic FLv | 35% | 20% |
| Lv5FU2 | 5.2% | 5.3% |
| Capecitabine | 26% | 31% |
| Nordic FLOX | 47% | 47% |
| FOLFOX | 24% | 30% |
| CAPOX | 2.9% | 0.6% |
| Nordic FLIRI | 30% | 57% |
| FOLFIRI | 18% | 38% |
| CAPIRI | 1.7% | 7.1% |
| Irinotecan | 0.6% | 6.5% |
| FOLFORINOX | 0.6% | 3.6% |
| **Adjuvant before mCRC** | 13% | 18% |
| **Targeted therapy** | | |
| Bevacizumab | NA | N=86 (51%) |
| EGFr | NA | N=59 (35%) |
| Bevacizumab+EGFr | NA | N=23 (14%) |
| **Number of cycles** (mean±SD) | | |
| Fluoropyrimidines* | 14.6±9.6 | 26.8±16.7 |
| Oxaliplatin* | 4.5±4.8 | 7.8±6.9 |
| Irinotecan* | 4.5±6.8 | 16.0±17.0 |
| **Targeted therapy (cycles)** (mean±SD) | | Bevacizumab 12.7±12.6 (N=86) |
| | | EGFr 9.0±7.7 (N=59) |
| | | Combination (N=23) |
| | | bevacizumab 10.1±10.3 |
| | | EGFr 16.2±14.2 |
| **Line EGFr TT** | | |
| **1st** | | N=36 (34%) |
| **2nd** | | N=34 (32%) |
| **3rd** | | N=28 (26%) |
| **4th** | | N=4 (4%) |
| **5th or later** | | N=3 (3%) |
| **Line bevacizumab** | | |
| **1st** | | N=30 (36%) |
| **2nd** | | N=15 (18%) |
| **3rd** | | N=19 (23%) |
| **4th** | | N=12 (15%) |
| **5th or later** | | N=7 (10%) |
